# Quantitative Risk Assessment of Oocyst Versus Bradyzoite Foodborne Transmission of *Toxoplasma gondii* in Brazil

**DOI:** 10.3390/pathogens12070870

**Published:** 2023-06-25

**Authors:** Sophie Zhu, Elizabeth VanWormer, Beatriz Martínez-López, Lílian Maria Garcia Bahia-Oliveira, Renato Augusto DaMatta, Pedro Souto Rodrigues, Karen Shapiro

**Affiliations:** 1 Department of Pathology, Microbiology and Immunology, School of Veterinary Medicine, University of California, Davis, CA 95616, USA; 2 School of Veterinary Medicine and Biomedical Sciences, University of Nebraska, Lincoln, NE 68588, USA; liz.vanwormer@unl.edu; 3 School of Natural Resources, University of Nebraska, Lincoln, NE 68588, USA; 4 Department of Medicine and Epidemiology, School of Veterinary Medicine, University of California, Davis, CA 95616, USA; 5 Department of Medicine, Institute of Medical Science, Universidade Federal do Rio de Janeiro, Macaé 27930-560, Brazil; 6 Laboratory of Cell and Tissue Biology, Universidade Estadual do Norte Fluminense, Campos dos Goytacazes, Rio de Janeiro 28013-602, Brazil

**Keywords:** quantitative risk assessment, foodborne pathogen, oocyst, *Toxoplasma gondii*, bradyzoite

## Abstract

*Toxoplasma gondii* is a globally distributed zoonotic protozoan parasite. Infection with *T. gondii* can cause congenital toxoplasmosis in developing fetuses and acute outbreaks in the general population, and the disease burden is especially high in South America. Prior studies found that the environmental stage of *T. gondii*, oocysts, is an important source of infection in Brazil; however, no studies have quantified this risk relative to other parasite stages. We developed a Bayesian quantitative risk assessment (QRA) to estimate the relative attribution of the two primary parasite stages (bradyzoite and oocyst) that can be transmitted in foods to people in Brazil. Oocyst contamination in fruits and greens contributed significantly more to overall estimated *T. gondii* infections than bradyzoite-contaminated foods (beef, pork, poultry). In sensitivity analysis, treatment, i.e., cooking temperature for meat and washing efficiency for produce, most strongly affected the estimated toxoplasmosis incidence rate. Due to the lack of regional food contamination prevalence data and the high level of uncertainty in many model parameters, this analysis provides an initial estimate of the relative importance of food products. Important knowledge gaps for oocyst-borne infections were identified and can drive future studies to improve risk assessments and effective policy actions to reduce human toxoplasmosis in Brazil.

## 1. Introduction

*Toxoplasma gondii* is a ubiquitous protozoan pathogen that has three infectious life stages: tachyzoites, bradyzoites in tissue cysts, and sporozoites in oocysts. The definitive hosts of *T. gondii*, domestic and wild felids, are the only animals that can shed environmentally resistant oocysts in their feces. Although most infections are asymptomatic, *T. gondii* can cause mild to severe disease in intermediate hosts, including humans [1]. Infection usually occurs via three pathways: (1) by eating meat from infected animals that harbor *T. gondii* tissue cysts, (2) by ingesting oocysts in contaminated soil, water, shellfish, or fresh produce [2], or (3) via vertical transmission of tachyzoites across the placenta after primary infection or maternal reinfection during pregnancy [3,4]. Since infection with *T. gondii* is lifelong, if a previously infected individual becomes immunosuppressed, *T. gondii* can reactivate and cause encephalitis, pneumonia, and even death [5]. Vertical transmission during pregnancy can cause congenital toxoplasmosis (CT), which can lead to spontaneous abortion, stillbirth, or ocular and developmental disease in the developing fetus. The current estimated global annual disease burden for CT is 1.15 million disability-adjusted life years (DALYs) [6]. Preventing exposure for high-risk groups (i.e., immunocompromised individuals and pregnant women) is vital because there are no human vaccines, nor effective treatments that can eliminate the chronic bradyzoite cysts from the tissues of infected individuals. 

Brazil is one of the most populous countries in South America, with approximately 213 million residents, and is characterized by high geographic heterogeneity, ranging from equatorial rainforests in the Amazon Basin to tropical savannahs in Central Brazil and even more diverse microclimates [7]. Toxoplasmosis disease burden is high in Brazil due to a combination of high environmental contamination, the presence of diverse, virulent genotypes of *T. gondii*, and poor or unreliable water quality [8]. The incidence of CT in Brazil ranges from 0.4 to 2 per 1000 births [6,9] and the prevalence of ocular toxoplasmosis can be as high as 17% in certain regions [10]. In Europe, meat-borne toxoplasmosis is perceived as a larger hazard (vs. oocyst ingestion) based on source attribution studies of pregnant women in the early 2000s, where 30–63% of seropositive cases were attributed to eating uncooked or cured meats, while only 6–17% of cases were attributed to soil contact [11]. In contrast, all reported outbreaks in Brazil with known or suspected routes of transmission since 2000 have been attributed to oocyst-based transmission [12]. Income inequality and disparity is a persistent social and economic issues, with additional consequences for health outcomes. Infection from oocysts may represent a higher proportion of total infections in Brazil due to the disproportionate exposure to waterborne pathogens for individuals from lower socioeconomic groups, who often have limited access to treated drinking water [13,14]. The combination of ecological, environmental, and socioeconomic factors makes Brazil a vital location to study food and waterborne *T. gondii* transmission. 

To prioritize intervention strategies, it is essential to identify the relative risk of exposure from different infection pathways. Various toxoplasmosis outbreaks have been reported worldwide, including in Canada and the United States; however, the majority of recent acute outbreaks have been reported in Brazil [15,16,17]. Common source attribution techniques used for outbreaks are epidemiological studies, outbreak investigation, expert elicitation, and risk assessment [18]. A recent expert elicitation by the WHO found that between 27 and 77% of acquired toxoplasmosis cases in South America are due to foodborne transmission [19]. Within foodborne transmission, few studies compare the importance of different food products such as meat and produce. Quantitative risk assessment (QRA), a method to estimate the potential risk of microbial exposure, has been applied to the meat-borne transmission of *T. gondii* in China, Italy, England, the Netherlands, and the United States; but to our knowledge, no studies have compared the relative risk of bradyzoite and oocyst-borne transmission from food [20,21,22,23,24,25]. QRA assessments utilize results from surveys, prevalence studies, and dose–response studies in order to integrate data into models that will be used to guide decision-making. Data on parasite prevalence are available for many meat products, but water, soil, and produce are matrices where *T. gondii* prevalence data are incredibly sparse; a recent systematic review found only 23 articles on the prevalence of *T. gondii* in fresh produce and 40 articles on *T. gondii* oocyst detection in water [26]. Of these studies, only six studies on water and two studies on produce were conducted in Brazil.

Given the limited knowledge surrounding the relative risk of oocyst foodborne *T. gondii* transmission, our main objective was to develop a Bayesian QRA model to estimate the risk associated with *T. gondii* exposure via ingestion of bradyzoites and oocysts from common foods in Brazil. Our aim was to characterize and compare the relative risk of oocyst vs non-oocyst infections and make management recommendations for transmission pathways of the highest concern to people in Brazil. 

## 2. Materials and Methods

**Figure 1:**
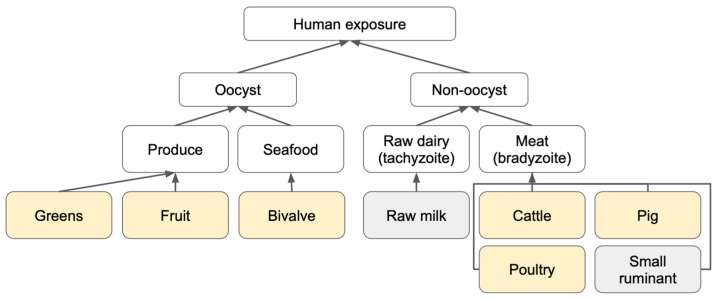
Summary of *T. gondii* transmission pathways by parasite stage and inclusion for consideration in the risk assessment model. Food groups evaluated in our models are highlighted in yellow, and food groups excluded based on lack of data are in gray.

**Figure 2:**
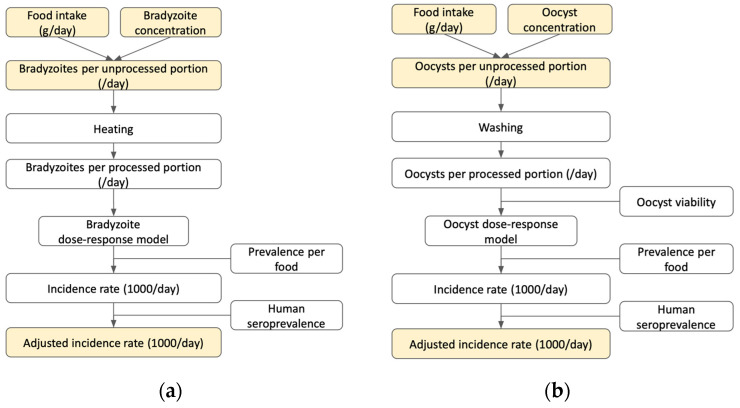
Conceptual framework for *T. gondii* infection via (**a**) bradyzoite-borne and (**b**) oocyst-borne food exposure in Brazil.

The stochastic Bayesian QRA that we applied for characterizing the risk of *T. gondii* exposure consists of four distinct steps: (1) hazard identification (Figure 1), (2) exposure assessment, (3) dose–response, and (4) characterization of risk from each transmission pathway (oocyst or bradyzoite) repeated for each food type (Figure 2). The final outcome of the model was an adjusted incidence rate per 1000 people per day, which accounts for immunity among Brazilians previously exposed to *T. gondii*. Given limited data on *T. gondii* across the various food matrices tested, a Bayesian risk assessment approach was selected to evaluate the probability of oocyst and bradyzoite *T. gondii* transmission in Brazil. Sensitivity and scenario analysis were also performed to evaluate a range of possible outcomes given different initial assumptions. All analyses were performed in R using the ‘rjags’ package [27] to interface between R and JAGS, a clone of BUGS (Bayesian analysis Using Gibbs Sampling). This framework can be adapted for future use when more data on regional and food-specific *T. gondii* prevalence are generated.

### 2.1. Hazard Identification

*T. gondii* foodborne exposure is broadly divided into two categories, non-oocyst and oocyst-borne (Figure 1). For foods contaminated with parasite stages that are not oocysts, we distinguish between meat-borne bradyzoite cyst infection from beef, pork, poultry, or ovine/caprine meat, and tachyzoite infections from unpasteurized dairy products. Oocyst-borne infections can result from the consumption of contaminated produce (fruits and vegetables), seafood (fish and/or shellfish), or water. Our study focused on the foodborne route to tease out food preferences that can be targeted for mitigating foodborne disease and thus did not include water as a potential source of exposure. Infections due to tachyzoites in raw dairy and bradyzoites from small ruminants were excluded from analysis due to the lack of dose–response and consumption data, respectively.

### 2.2. Exposure Assessment

**Figure 3:**
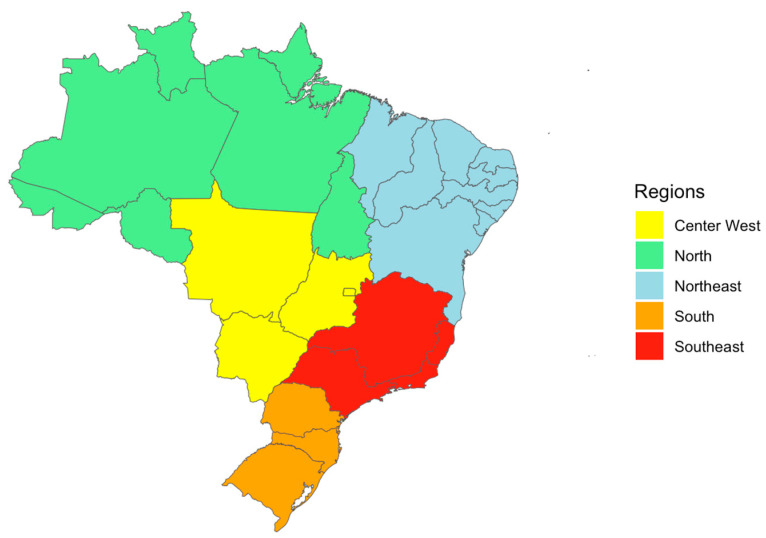
The five major regions of Brazil as classified by the Brazilian Institute of Statistics (IBGE). States are grouped based on similar geographical and socioeconomic characteristics, though there is still high heterogeneity both between and within regions.

Data on average daily consumption of specific foods that were included in our study were obtained from the 2017–2018 edition of Pesquisa de Orçamentos Familiares (POF, Family Budget Research) [28], which is administered by the Brazilian Institute of Geography and Statistics (IBGE). The data (mean grams consumed/day) focus on a subset of the Brazilian population aged 10 years and older and encompasses differences in urban and rural areas across all Brazilian states. The twenty-six states and one federal district are commonly grouped into five regions based on geographic, social, and economic factors as used by the IBG:; north, northeast, center-west, southeast, and south (Figure 3).

#### 2.2.1. Food Intake Quantity (g/day)

##### Bradyzoite Exposure—Meat

The daily average per capita consumption of specific meat products from the 2017–2018 IBGE POF survey was grouped for analysis purposes under the source animal (Table S1). Three broad categories were used; beef, pork, and poultry. Lamb and mutton were not incorporated into the model due to a lack of data on consumption from IBGE, and a relatively low reported level of consumption (1.26 kg/year) according to the Organization for Economic Co-operation and Development [29].

##### Oocyst Exposure—Fresh Produce

For this study, we focused on fruits and vegetables that were previously associated with reports of *T. gondii* infections, i.e., salad mix, leafy greens, and açai berries [30,31]. Salad mix, leafy greens, and cabbage were combined under total “greens” consumption to reduce the number of categories in modeling. Total consumption of either greens or fruit was considered rather than consumption of individual products (mixed salad, lettuce).

##### Oocyst Exposure—Seafood

Shellfish and fish can serve as mechanical vectors of *T. gondii* oocysts, passively concentrating oocysts in tissues or the gastrointestinal tract without serving as a true intermediate host for the parasite [32]. As fish digestive tracts or gills (where oocysts may be present) are generally removed before eating, the risk of oocyst exposure from fish is estimated to be lower than that of shellfish that are usually eaten whole and sometimes raw. Shellfish consumption is not evenly distributed across Brazil, nor was consumption data available for shellfish from the IBGE. To demonstrate how our model can be applied to a food product with limited data and to assess the potential role of raw shellfish consumption in foodborne toxoplasmosis risk, we obtained Pacific and native oyster production data from the state of Santa Catarina, which is the only state with industrial scale shellfish production, and converted it to estimated consumption for the southern region of Brazil (Table S2). Due to the limited data on shellfish consumption and oocyst concentration, we only assessed shellfish as a food source in scenario-specific analysis, not in the baseline model.

#### 2.2.2. Parasite Concentration

The estimate of bradyzoite cyst concentration per gram of meat from infected animals was obtained from a study of naturally infected sheep in the Netherlands; although different hosts have different propensities to form cysts, this is one of the only published studies of cyst concentration in livestock and these estimates were used by other assessments of meat-borne *T. gondii* transmission [33]. The log10-transformed concentration of bradyzoites in an infected animal was described by a generalized beta distribution with shape α1 of 6.5, α2 of 5.7, a minimum of 0, and a maximum of 6.8 (mean 3.6; 3981 bradyzoites per 100 g) as used by Opsteegh et al. and Deng et al. [23,24]. Since the JAGS framework does not support the generalized beta distribution by default, we used normalization to convert this to a beta distribution N1.1, where N1 is the original distribution, a is the minimum of N1, and b is the maximum of N1 [34].

$$N1.1\sim \frac{N1-a}{b-a}$$

$$N1.1=\frac{N1}{6.8}=\left(\frac{6.5,\;5.7,\;0,\;6.8}{6.8}\right)$$

N1.1 = beta(0.955, 0.838) × 6.8

Samples drawn from this distribution were then multiplied by 6.8 (the maximum concentration from the generalized beta distribution N1) to convert the values back into the proper log10-transformed concentration (Table 1). 

As no studies of oocyst concentration on produce in Brazil exist, *T. gondii* oocyst concentration on fruits and vegetables was estimated using data from Spain and Portugal [35]. The theoretical distribution according to the skewness–kurtosis graph from the R package ‘fitdistrplus’ showed the data were beta distributed [47]. Oocyst concentrations for fruits and vegetables were transformed into a proportion by dividing by the maximum concentration per gram of produce plus 0.1 (179.9 oocysts/g + 0.1 = 180) in order to be fit to a beta distribution. The transformed data followed a beta distribution with shape 1 = 0.26 and shape 2 = 0.70, which we used for both fruit and greens due to the small sample size for both types of produce. Samples drawn from this distribution were multiplied by 180 (maximum concentration from N4 + 0.1) to convert the values from proportions back into concentrations. To date, there have been no reports in the literature that quantified oocyst concentrations in harvested shellfish; however, experiments in our laboratory [48] found that the limit of detection was about five *T. gondii* oocysts per oyster. Another study in São Paulo demonstrated that the limit of detection by nested PCR was 100 oocysts [49]. Therefore, these values were used as a lower and a (conservative) upper limit of detection for calculating the range of oyster *T. gondii* oocyst burden. Assuming that the average edible weight of Pacific oysters (*Crassostrea gigas*), the primary shellfish species produced in Brazil, is ~20 g [50], the maximum concentration of oocysts would be 100/20 g or 5/g; because we did not have data for distribution fitting, we used a uniform distribution with range 0.1–5 oocysts per gram of oyster consumed for this parameter to encompass a broad range of potential values.

#### 2.2.3. Parasites per Unprocessed Portion

The number of parasites per unprocessed portion of each food item tested was calculated with the following equations from Table 1:

N2∼Poisson (*λ* = C_meat_ × (10^N1^/100))

where the number of bradyzoites (N2) follows a Poisson distribution with *λ =* the amount of each type of meat consumed multiplied by 10 to the power of bradyzoite concentration divided by 100. 

The number of oocysts (N5) was also assumed to be Poisson distributed with *λ* calculated as the product of the amount of produce consumed multiplied by produce oocyst concentration.

N5∼Poisson (*λ* = C_produce_ × N4)

#### 2.2.4. Parasites per Processed Portion

To account for the loss of bradyzoite infectivity in cooked meat, the number of bradyzoites in infected meat that was cooked was calculated using the methods of Deng et al., who multiplied the number of bradyzoites in unprocessed meat by a temperature reduction factor [24], RF based on cooking temperatures in US households (Table 1). 

Similarly, to account for the physical removal of oocysts from washed produce, the number of oocysts on washed produce was calculated in a similar manner using N6: N5 X W(T), where the number of oocysts remaining on washed produce (N6) was equal to the product of oocysts on unwashed produce (N5) times the proportion of oocysts remaining after washing, W(T). As there are no previous studies to our knowledge that have described the effect of washing on oocyst removal, we used washing data for *Cyclospora cayetanensis* (a related protozoan parasite) from berries [36]. Sporulated *T. gondii* oocysts are 10–12 µm, which is similar to the size of *Cyclospora* oocysts (7.5–10 µm). The proportion of *Cyclospora* oocysts remaining after washing with water for 1 min was fit to a distribution using the package ‘fitdistrplus’ [47] and followed a beta distribution with shape α1 = 1 and α2 of 0.57. No reduction factor (washing) or heat inactivation parameter was used for shellfish because oysters are often eaten whole and raw. 

### 2.3. Dose–Response Assessment

The dose of potentially infective *T. gondii* oocysts consumed through food was calculated by multiplying the amount of food matrix consumed (grams) by *T. gondii* stage concentration (per gram) and contamination prevalence for each food product. Dose–response equations from previous *T. gondii* bradyzoite-borne and oocyst-borne risk assessments on mice and rats were used as no human dose–response experiments exist [23]. The probability that a single bradyzoite will lead to infection, also known as single hit probability of infection (r1), is r = 0.001535 (Table 1). The single hit probability of oocysts used in dose–response equations was obtained from a recent study that used scaled and combined mouse, rat, and pig data [37]. A bradyzoites viability parameter in meat products was not considered because all meat was assumed to be prepared from fresh; freezing meat for an extended period of time can inactivate bradyzoites [51]. In contrast, not all *T. gondii* oocysts that are present on food matrices are sporulated or viable, and there are no studies reported to date that have tested for oocyst viability on produce in field studies. As a conservative estimate for oocyst viability, we assumed that only 50% of oocysts present in consumed foods were viable in our baseline model. The probability of illness from bradyzoites and oocysts was calculated with P_1_ and P_2_, respectively (Table 1).

### 2.4. Risk Characterization

#### 2.4.1. Prevalence of Contamination by Food Product

*T. gondii* contamination across different types of food items varies geographically, so we collected estimates specific to Brazil whenever possible, and fit data to distributions to capture variability across studies (Table S2). When prevalence estimates in Brazil were not available, we used data from other countries, as was the case for fruit. 

#### 2.4.2. Adjusted Incidence Rate

Many Brazilians have previously been infected with *T. gondii* and are likely to be immune to new infections in our analysis. To account for this, we calculated an adjusted incident exposure rate that accounts for prior immunity. Seroprevalence is highly variable within Brazil, but we obtained a pooled estimate of 28.6% (17,535/61,283) for the average seroprevalence of *T. gondii* in Brazil from a recent meta-analysis [52]. Most studies from the meta-analysis focused on women of reproductive age, but for the purposes of our study, we assumed this prevalence was similar in the general population [52]. The number of infections in each region of Brazil was calculated by multiplying the exposure incidence rate/1000 people per day by a factor of 0.714 to estimate the number of new infections per year in the susceptible population.

### 2.5. Data Analysis

Analysis was implemented in R version 4.2.2 using the packages ‘rjags’ and ‘coda’ [27,53]. The mean number of cases of oocyst or bradyzoite-borne toxoplasmosis from each source was calculated for the general population with 4 chains, 10,000 samples for adaptation, 50,000 iterations, and a burn-in interval of 5000. Autocorrelation and model convergence were assessed by looking at the trace and gelman plots (Figures S3 and S4).

### 2.6. Sensitivity and Scenario Analysis

Consumption amount, food prevalence, cooking temperature (meat), or washing (produce) were varied in sensitivity analysis of each parameter from −50 to +50% of the baseline estimate. Mean total cases were calculated using consumption quantities averaged over all five regions (Table S1). A total of four scenarios were evaluated: (1) a lower concentration of bradyzoites in beef (1/100) compared to other meat products, (2) a produce washing efficiency that increases oocyst removal by 25%, (3) a decrease in oocyst viability by 50%, and (4) incorporation of shellfish as another food source of infectious oocysts (Table 2). Many *T. gondii* experts do not believe that beef is a significant source of exposure due to the difficulty in isolating viable infectious parasites from cattle muscle tissues [39,54]. The first scenario explores one potential mechanism which may explain the lower source attribution of beef by lowering bradyzoite concentration. In scenario two, we explored how improved washing efficiency alone could reduce parasite exposure by highlighting a food preparation practice that can be easily implemented by consumers. In scenario three, we assumed that oocyst viability was half of the baseline model (50% viable), or 25% overall oocyst viability. In scenario four, shellfish consumption was added based on production patterns and dietary habits from correspondence with collaborators in the state of Rio de Janeiro (P. Souto Rodrigues, R. DaMatta, and L. Bahia-Oliveira).

## 3. Results

**Figure 4:**
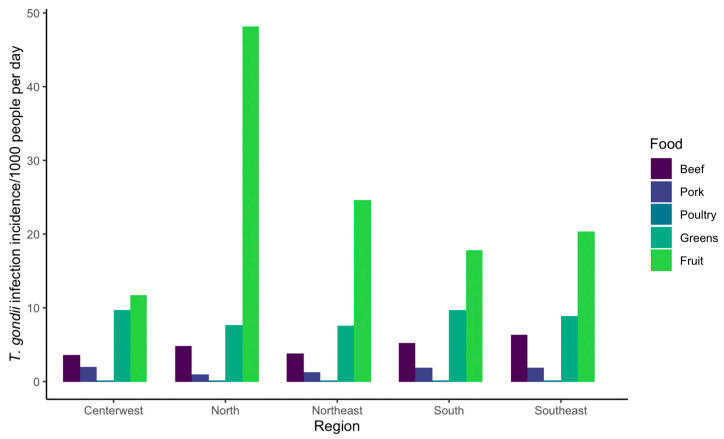
Baseline results for risk analysis estimating the mean oocyst and bradyzoite foodborne toxoplasmosis incidence/1000 people per day in five regions of Brazil.

The mean incidence of foodborne *T. gondii* infections per 1000 people per day that were estimated in our risk analysis are summarized in Table 3 and Figure 4. Across the five regions of Brazil, the estimated range of mean incidence was higher for oocyst-borne infection (leafy greens 7.58–9.68 and fruit 11.76–48.13, respectively) as compared with bradyzoite-borne infections from meat (beef 3.58–6.32, pork 0.97–2.03, and poultry 0.11–0.17). Overall, the estimated incidence of *T. gondii* infection was highest in the north, and oocyst-borne infections from fruit contributed the most to the risk of foodborne infection in this region. Risk estimates for oocyst-borne infections from greens and fruit had very large credible intervals (CI); for example, the incidence of infection per 1000 people from fruit in the north region of Brazil was 48.13, but the 95% CI ranged from 0 to 314.2 (Table 3).

### 3.1. Sensitivity Analysis

We varied parasite concentration, consumption quantity, *T. gondii* prevalence in food, and heating or washing efficiency to evaluate the effects of these parameters on the estimated incidence of foodborne infection. Our sensitivity analysis demonstrated that heating and washing efficiency were the most important variables affecting our incidence estimates of *T. gondii* infection from bradyzoites and oocysts, respectively (Figure S1). 

### 3.2. Scenario Analysis

In addition to the baseline model that estimated *T. gondii* infection incidence by region, several scenarios were chosen to evaluate the role of specific foods and processing methods that may be of high interest to food producers and/or consumers (Table 2). In the first scenario, reducing the concentration of bradyzoites in beef compared to other meat types reduced the estimated incidence from beef consumption compared to the baseline model (−99%). When oocyst washing efficiency was improved by 25%, the estimated infections per 1000 persons per day were greatly reduced from both greens (−35%) and fruit (−40%).

A reduction in the number of oocysts that were viable on produce also reduced infections from greens (−14%) and fruit (−10%), and thus total infections; however, this reduction was less pronounced as compared with the oocyst washing efficiency scenario. Finally, we explored how other food sources such as shellfish can play an important role in local risk for foodborne T. gondii exposure. Although the average estimated consumption of shellfish was low, incorporating this seafood as a product did increase total incidence slightly (+0.6%) (Figure S2).

## 4. Discussion

In this study, we present the first risk analysis model designed to quantify and discriminate the risk of oocyst and bradyzoite foodborne transmission in Brazil. Exposure assessments for oocysts are especially needed as a recent review of the literature found that out of seven toxoplasmosis outbreaks that occurred in Brazil between 2000 and 2018, 42.8% were attributed to meat and 28.6% were attributed to produce, highlighting the importance of oocyst exposure [12]. The remainder of recent outbreaks were due to water (14.3%) and soil (14.3%), which further highlights the importance of oocyst exposure to human infection. Congenital and acute toxoplasmosis disease burdens are both incredibly high in Brazil [8,9,10,55], and quantitative assessment tools are necessary in order to improve food safety practices that can help reduce the risk of exposure.

Estimated incidence rates for *T. gondii* exposure were simulated for five regions of Brazil to identify the relative importance of different foods as sources of infection in regions that may have different dietary habits. Beef contributed the most to meat-borne infection in all regions (based on an assumption in the baseline model of equal bradyzoite cyst burden in cattle and sheep), which agrees with the findings of a Dutch QRA but not with a more recent QRA performed for China [23,24]. We acknowledge that this finding depends heavily on the baseline model assumptions, which may be appropriate for pork and poultry but less so for beef. These differences may depend heavily on food consumption preferences between countries; even within our analysis, the estimated incidence from a given food group could be 1.5–2 times higher between the lowest and highest regions (Table 3). Regional heterogeneity of infections was also present in a recent retrospective analysis of infant mortality associated with congenital toxoplasmosis in Brazil, suggesting that regional exposure risk differences can play a role in pathogen transmission [56]. Predicted infections from oocyst exposure through the consumption of fresh produce (i.e., fruits and greens) were up to 100 times greater than infections from eating meat (Table 3), which may be an overestimate of the actual relative importance of produce due to assumptions made in our baseline model on oocyst load and viability as discussed below.

Sensitivity analyses demonstrated that oocyst concentration on fresh produce had a substantial effect on estimated risk. Our estimates of this parameter were from a single study in Portugal and Spain that quantified the number of oocysts on fresh leafy greens and berries from local suppliers/markets [35]. We pooled data for *T. gondii* oocyst count on greens and fruit due to the limited number of samples, which may not accurately capture differences in oocyst load among different types of produce. The presence and amount of parasite contamination can vary dramatically across geographic regions and can be affected by produce source, washing prior to sale, storage conditions, vendor knowledge of food safety, as well as produce type [57,58,59]. Produce contamination by *T. gondii* during cultivation or transport cannot be controlled by consumers, so this is an aspect of food production and processing that could be more tightly regulated by improvements to food safety practices and guidelines. In tandem with production level changes, improved education and awareness can lead consumers to alter their individual food consumption and preparation practices in order to reduce *T. gondii* oocyst exposure. 

Under experimental conditions, a single oocyst can cause *T. gondii* infection in an intermediate host such as a rodent or pig, though the exact infectious dose for humans is unknown [38,60]. Additionally, not all oocysts in the environment are viable and infective, or able to lead to excystation, cell invasion, and replication in a host. The proportion of oocysts that are viable or infective on produce was another parameter where we lack robust quantitative estimates. In soil, oocyst viability can range from 7.4% in dry conditions to 43.7% in damp conditions after 100 days [61]. As many vegetables are grown in soil, these values could be used to inform estimates of oocyst concentration on leafy greens. New detection methods for distinguishing between viable and non-viable oocysts in water exist, but many of these techniques are not yet widely available or used across studies that estimate *T. gondii* oocyst contamination in foods [62]. Like Deng et al. we assumed a baseline viability of 50% [63] but recognize that this assumption may overestimate the true oocyst viability/infectivity on fruits, vegetables, and within shellfish tissues, and thus the estimated incidence of oocyst-borne infections in our model. 

Washing produce in order to remove oocysts was the most influential variable for oocyst-borne infections in our sensitivity analyses, but similar to oocyst concentration, there were few published sources that we could use in determining accurate distributions for model inputs. The study that we referenced looked at the percentage of parasites (*Cyclospora cayetanensis*) removed from blueberries and raspberries using different methods such as washing in running water, washing with vinegar, and using a salad spinner after washing [36]. As the size and surface properties of different parasite oocysts can vary, *T. gondii* oocysts may actually be more or less resistant to removal by washing compared to *Cyclospora*. Variables such as berry type, washing duration, tap water quality, water infrastructure quality, and parasite type could all change the proportion of oocyst removal due to washing. We assumed that most households in Brazil would wash their produce with tap water but testing how washing and other treatments such as ultraviolet light disinfection (UV) can remove or inactivate *T. gondii* oocysts on produce can enhance our understanding of risk in realistic settings. The exact method of parasite treatment may be more or less relevant depending on the location of the study, as methods such as UV may be less realistic for widespread application in low- and middle-income countries.

We recognize that *T. gondii* prevalence in different foods will vary across regions within a country or even locally. Brazil is the largest country by land mass and population size in South America, and a handful of studies cannot cover the breadth of variation in *T. gondii* contamination patterns across different food matrices and subsequent *T. gondii* exposure. For example, *T. gondii* seroprevalence in the state of Minas Gerais can range from 2.7 to 31.4%, a 10-fold difference [64,65]. Regional or individual food preparation, consumption preferences, and socioeconomic status can also alter exposure risk [52,66]. In addition to heterogeneity in regional parasite prevalence in foods, the consumption data we used from IGBE is supposed to be representative of consumption patterns by region, but these values are a population average. The results presented in this study describe average relative risk, not absolute risk, and are better suited to guide general public health recommendations rather than individual changes in food preferences. 

We explored structural assumptions of our model in scenario analysis, namely reducing bradyzoite concentration in beef, increasing washing efficiency for produce, lower oocyst viability, and inclusion of shellfish as an additional food group. Although beef was the primary source of toxoplasmosis from meat, we acknowledge that this may overrepresent the actual risk of infection due to the low numbers of viable bradyzoites that are isolated from beef compared to meat from other livestock species [39]. Similar to Deng et al., we found that when the concentration of *T. gondii* cysts in beef was reduced to 1/100 of other meat, toxoplasmosis incidence decreased to be more on par with pork and poultry [24]. Both increased oocyst removal from washing and an assumption of lower oocyst viability reduced estimated toxoplasmosis incidence, though washing was more effective (Figure S2). This suggests that more empirical information on viability and washing may need to be gathered and that in the meantime washing with clean water sources (free of *T. gondii* oocyst contamination) could be prioritized in the dissemination of proper food safety guidelines to consumers. Shellfish consumption is a risk factor for toxoplasmosis [67] as they are generally consumed whole and may be eaten raw, putting consumers at a greater risk of exposure to *T. gondii* compared to foods that are treated to remove or inactivate parasites. We explored the role of shellfish consumption preferences in the south of Brazil, the main region where shellfish is produced commercially. As shellfish consumption only represented a small fraction of the food consumed on a daily basis (0.185 g/person/day Table S3), this food accounted for 0.6% of infections in scenario four, which was roughly the same percentage as poultry (0.2%). Though the predicted risk of infections from shellfish in our model was small, production of shellfish in Brazil has increased in the last decade as its popularity has grown among consumers. If shellfish becomes a more lucrative and sought-after product within and outside of southern Brazil, infections from this food type may become more common. There are no official reports of shellfish consumption because current production and consumption are still low; however, this example illustrates how we can use modeling to explore relative patterns and potential scenarios as food preferences change or as more data are produced from ongoing research.

Oocyst genotype and virulence were not accounted for in our model, but it is important to note that not all *T. gondii* genotypes may be equally efficient at causing severe symptoms in humans [68]. As South America has high *T. gondii* genetic diversity, there are many opportunities for novel and repeated exposure to virulent genotypes [69,70]. Prior exposure to *T. gondii* in Brazil is common; up to 70% of pregnant women are seropositive in regional surveys [52]. Infection with one genotype may not be protective against future *T. gondii* infections with different parasite genotypes; mixed infections in humans can lead to systemic illness [4,71,72]. Thus, a level of immunity that completely protects against reinfection in the Brazilian population is not likely to be reached due to the diversity of circulating strains. This may be one of the limiting intrinsic factors of our model. 

Though previous epidemiological studies in Brazil have highlighted the importance of waterborne transmission, we were unable to evaluate water as a source of *T. gondii* oocysts due to the limited data on *T. gondii* prevalence and oocyst concentration in water. Additionally, we considered regional patterns of food consumption, whereas water is essential and has less geographic variability in consumption. The ability of contaminated water to cause large-scale outbreaks in Brazil and elsewhere cannot be underestimated [13,14,15]. Numerous waterborne outbreaks have been reported in Brazil since 1966, including a 2018 outbreak with at least 2270 suspected cases, which was the largest oocyst-associated outbreak reported to date [17]. One of the reasons that waterborne transmission is so common is variability in the quality of drinking water available to many Brazilians on average [73] and huge disparities in the availability and quality of clean water in Brazil across socioeconomic levels [74]. Aside from a lack of knowledge of proper food safety, unreliable or nonexistent access to treated water can put individuals from poorer socioeconomic backgrounds at a higher risk for *T. gondii* exposure. Mitigating transmission in poorer and/or rural areas of Brazil may require greater attention and resources in order to equitably address disease prevention. Improving existing infrastructure to increase access to clean, potable water can have cascading benefits to *T. gondii* transmission as a whole by reducing waterborne exposure and allowing for increased removal of oocysts from produce when clean tap water is used for washing food.

## 5. Conclusions

We developed a novel QRA model for the relative comparison of oocyst and bradyzoite-borne *T. gondii* infections from food in Brazil. Predicted infections from produce were roughly 8–13 times higher than infections from meat sources across different regions. Modeling studies which identify potential knowledge gaps are critical for exploring appropriate risk mitigation at different food production and preparation steps. For example, in addition to sampling food products to obtain *T. gondii* contamination prevalence, studies that identify sources of oocyst contamination at farms, produce processing, or transport can be conducted in tandem. Oocyst-borne transmission of *T. gondii* should be given greater attention and resources for intervention and management, and avenues for further research include investigations on oocyst concentration and viability, oocyst removal and inactivation methods, contamination prevalence, and characterization of different parasite genotypes in common food products. As more data on these variables, especially contamination prevalence and oocyst removal methods become available, we can continue to use this modeling framework in Brazil and in other countries to obtain more accurate estimates of *T. gondii* infection risk from oocyst-contaminated foods as compared with meat-borne infections.

## Figures and Tables

**Table 1:** Model parameters and equations used in Bayesian QRA of foodborne toxoplasmosis in Brazil.

Variable	Equation/Distribution/Value	References
**Exposure assessment bradyzoite**		
N1: n log10-transformed bradyzoites/100 g unprocessed meat	N1∼Beta general (shape 1 = 6.5, shape 2 = 5.7, min = 0, max = 6.8) N1 transformed (N1.1) = Beta(0.955, 0.838) × 6.8	[23]
N2: n bradyzoite consumed/meat type	N2∼Poisson (*λ* = C_meat_ × (10^N1×6.8^/100))	[23]
C: food consumption amount (g)	Table S1	IBGE [28]
N3: n bradyzoites after home cooking per consumed portion	N3 = N2 × RF(T)	[23]
RF(T): temperature reduction factor	RF(T) = D(T)/D(T0)	[23]
D(T): dose of bradyzoites	D(T) = −ln(1 − P0(T))/r0	[23]
P0(T): probability of infection in mice	P0(T) = 1/(1 + e^^−(44.181−0.834×T)^	[24]
r0: *T. gondii* infection probability of a single bradyzoite in mice	r0 = 0.011	[24]
T: temperature C	T~Laplace (m = location, s = dispersion) Pork m = 71.11, s = 9.88 Beef and sheep m= 71.11, s = 9.82 Poultry m = 75.56, s = 9.31	[23]
T0: temperature before cooking (C)	T0 = 25	[23]
**Exposure assessment oocyst**		
N4: n oocysts/g unprocessed produce	N4~Beta (shape 1 = 0.105, shape 2 = 0.702)	Fitted data from Marques et al. [35]
N5: n oocysts consumed/produce type	N5∼Poisson (*λ* = Cproduce × N4 × 180)	[23]
N6: n oocysts after washing W(T): washing	N6 × W(T) W(T)~Beta (1, 0.57)	Distribution fit from Temesgen et al. 2021 [36]
**Dose–response**		
P_1_: probability of human infection (per meat type in one day)	P_1_ = 1 − e(−r1 × N3)	[37]
r1: probability of single bradyzoite initiating *T. gondii* infection in humans	r1 = 0.001535	[37]
P_2_: probability of human infection (per produce type in one day)	P_2_ = 1 − e(−r2 × N6)	[38]
r2: probability of single oocyst initiating *T. gondii* infection in humans	r2 = 0.46	[38]
**Risk characterization**		
P_3_: probability of infection through consumption of food products in human population (/meat or produce type per day)	P_3_ = P_1_ or P_2_ × P_food_ × (1 − P_human_)	[23]
P_food_: prevalence per food type. Overall averaged prevalences for the entirety of Brazil were used due to the low number of available studies.	P_food_: Supplemental Table S2	[39,40,41,42,43,44,45,46]
P_human_: seroprevalence of human population	P_human_: Unif (0.215, 0.974)	[8]

**Table 2:** Description of scenarios assessed in QRA model of foodborne toxoplasmosis burden in Brazil.

Scenarios	Definitions
Baseline model	Baseline consumption of food products averaged over all five regions of Brazil to compare against other scenarios
(1) Lower bradyzoite concentration in beef	Bradyzoite concentration for beef lowered to 1/100 that of other meat species to reflect lower estimated tissue cyst burden in cattle
(2) Improved washing efficiency	Washing oocysts off of produce increased by 25% from baseline
(3) Decreased oocyst viability	Oocyst viability reduced by 50% (25% viability overall)
(4) Shellfish	Oyster consumption added as another source of oocyst foodborne transmission due to its growing popularity. Production data were obtained for the South of Brazil and converted to consumption quantity (g) (Table S3).

**Table 3:** Baseline model with estimated incidence of foodborne toxoplasmosis per 1000 people, 95% credible interval [CI], and percentage of total infections by region.

	North		Northeast		Center-West		Southeast		South	
Food	Mean (95% CI)	%	Mean (95% CI)	%	Mean (95% CI)	%	Mean (95% CI)	%	Mean (95% CI)	%
Beef	4.81 (1 × 10^−6^, 46.1)	7.8	3.78 (0, 48)	10.1	6.32 (4 × 10^−8^, 59.4)	16.9	5.19 (0, 53.4)	14.9	3.58 (8 × 10^−7^, 39.6)	13.2
Pork	0.97 (0, 12.7)	1.6	1.28 (0, 16.3)	3.4	1.91 (0, 20.3)	5.1	1.91 (0, 20.7)	5.5	2.03 (0, 23.6)	7.5
Poultry	0.13 (1 × 10^−7^, 1.3)	0.2	0.17 (0, 1.8)	0.5	0.11 (0, 1.1)	0.3	0.15 (0, 1.36)	0.4	0.11 (0, 1.1)	0.4
Greens	7.69 (0, 60.2)	12.5	7.58 (0, 57.4)	20.3	8.86 (0, 71.9)	23.6	9.68 (0, 70.4)	27.9	9.67 (0, 80.3)	35.6
Fruit	48.13 (0, 314.2)	77.9	24.62 (0, 259.5)	65.7	20.31 (0, 179.5)	54.1	17.85 (0, 198.1)	51.3	11.76 (0, 118.1)	43.3
Total	61.73		37.43		37.51		34.78		27.15	

## Data Availability

No new data were created or analyzed in this study. Data collected from other published materials are available in a consolidated form in supplementary materials.

## Supplementary Materials

The following supporting information can be downloaded at: https://www.mdpi.com/article/10.3390/pathogens12070870/s1, Table S1: Average daily consumption of food products (g) by region, data from 2017 to 2018 IGBE POF survey [28]; Table S2: Prevalence of *T. gondii* in food by product type; Table S3: Declared federal production of oysters in Santa Catarina, Brazil in 2020; Figure S1: Sensitivity analysis visualized through tornado plots; Figure S2. Scenario analysis results for putative scenario of interest; Figure S3. Sample Gelman plots for Bayesian QRA model; Figure S4. Sample trace plots for Bayesian QRA model. Supplemental Code: Code used for QRA model analysis using rjags and coda. References [75,76,77,78,79] are cited in the supplementary materials.
