# Supplementary material for: Quantitative Risk Assessment of Oocyst Versus Bradyzoite Foodborne Transmission of Toxoplasma gondii in Brazil
Source: Pathogens. 2023 Jun 25;12(7):870. doi: 10.3390/pathogens12070870 (PMC10384504; doi:10.3390/pathogens12070870)
Supplement: Supplementary file 1 [file pathogens-12-00870-s001.zip › pathogens-2446130-supplementary.pdf]

Table S1. Average daily consumption of food products (g) by region, data from 2017-2018 IGBE POF survey [28]

Stage	Food	North	Northeast	Southeast	South	Center West	Average (used in sensitivity and scenario analysis)
Bradyzoite	Bovine	58.6	41.5	48.4	50.8	80.7	56
	Porcine*	15.3	15.1	20.3	29.5	31.5	22.34
	Poultry	51.8	58.7	44.9	37.4	35.8	45.72
Oocyst	Greens	12.3	14	28.8	22.5	37.7	23.06
	Fruit	45.4	1.6	1.1	0.4	2.6	10.22

*Porcine meat included pork as well as specific pork derivatives such as linguiça (smoke-cured pork sausage), mortadella (heat-cured pork), and ham [75,76].

References

75. Dias, R.A.F.; Navarro, I.T.; Ruffolo, B.B.; Bugni, F.M.; de Castro, M.V.; Freire, R.L. *Toxoplasma gondii* in fresh pork sausage and seroprevalence in butchers from factories in Londrina, Paraná State, Brazil. *Rev. Inst. Med. Trop.* **2005**, *47*, 185–189.
76. Herrero, L.; Gracia, M.J.; Pérez-Arquillué, C.; Lázaro, R.; Herrera, A.; Bayarri, S. *Toxoplasma gondii* in raw and dry-cured ham: The influence of the curing process. *Food Microbiol.* **2017**, *65*, 213–220.

Table S2. Prevalence of *T. gondii* in food by product type

Food	Distribution	Sources
Meat - Bovine	Beta ~ (0.74, 1.90)	Brazil [39]
Meat - Porcine	Beta ~ (1.12, 3.87)	Brazil [40]
Meat - Poultry	Beta ~ (1.19, 2.14)	Brazil [41]
Produce - Greens	Beta ~ (0.7, 19.9)	Brazil [42-45]
Produce - Fruit	Beta ~ (0.66, 3.56)	Columbia [46], Spain/ Portugal [34]
Seafood – Bivalves*	Beta ~ (24.7, 1112.8)	Brazil [13]

* Values used in scenario 4 only

References

13. Minuzzi, C.E.; Fernandes, F.D.; Portella, L.P.; Bräunig, P.; Sturza, D.A.F.; Giacomini, L.; Salvagni, E.; Ribeiro, J.D.S.; Silva, C.R.; Difante, C.M.; et al. Contaminated water confirmed as source of infection by bioassay in an outbreak of toxoplasmosis in South Brazil. *Transbound. Emerg. Dis.* **2021**, *68*, 767–772.
34. Otieno, J. From the Classical Beta Distribution to Generalized Beta Distributions. Master's Thesis, University of Nairobi, Nairobi, Kenya, 2008.
39. Dubey, J.P.; Murata, F.H.A.; Cerqueira-Cézar, C.K.; Kwok, O.C.H.; Yang, Y.R. Public health significance of *Toxoplasma gondii* infections in cattle: 2009–2020. *J. Parasitol.* **2020**, *106*, 772–788.
40. Dubey, J.P.; Cerqueira-Cézar, C.K.; Murata, F.H.A.; Kwok, O.C.H.; Hill, D.; Yang, Y.; Su, C. All about *Toxoplasma gondii* infections in pigs: 2009–2020. *Vet. Parasitol.* **2020**, *288*, 109185.
41. Dubey, J.P.; Pena, H.F.J.; Cerqueira-Cézar, C.K.; Murata, F.H.A.; Kwok, O.C.H.; Yang, Y.R.; Gennari, S.M.; Su, C. Epidemiologic significance of *Toxoplasma gondii* infections in chickens (*Gallus domesticus*): The past decade. *Parasitology* **2020**, *147*, 1263–1289.
42. Marchioro, A.A.; Tiyo, B.T.; Colli, C.M.; de Souza, C.Z.; Garcia, J.L.; Gomes, M.L.; Falavigna-Guilherme, A.L. First detection of *Toxoplasma gondii* DNA in the fresh leafs of vegetables in South America. *Vector Borne Zoonotic Dis.* **2016**, *16*, 624–626.
43. Ferreira, F.P.; Caldart, E.T.; Freire, R.L.; Mitsuka-Breganó, R.; de Freitas, F.M.; Miura, A.C.; Marenze, M.; Martins, F.D.C.; Urbano, M.R.; Seifert, A.L.; et al. The effect of water source and soil supplementation on parasite contamination in organic vegetable gardens. *Rev. Bras. Parasitol. Vet.* **2018**, *27*, 327–337.
44. Pinto-Ferreira, F.; Mitsuka-Breganó, R.; Monica, T.C.; Martins, F.D.C.; de Matos, R.L.N.; Mareze, M.; de Souza Lima Nino, B.; Narciso, S.G.; Freire, R.L.; Navarro, I.T. Investigation and environmental analysis of samples from outbreak of toxoplasmosis at research institution in Londrina, Paraná, Brazil, 2016. *Braz. J. Vet. Parasitol.* **2019**, *28*, 518–521.
45. Perim, L.V.; Custódio, N.C.C.; de Castro Vieira Lima, V.; da Igreja, J.A.S.L.; de Sousa Mendes Moreira Alves, D.; Storchilo, H.R.; Gomes, A.R.; de Castro, A.M.; da Costa, W.L.G.; Rezende, H.H.A. Occurrence of parasites in salads in restaurants in Aparecida de Goiânia, Goiás, Brazil. *Rev. Patol. Trop. J. Trop. Pathol.* **2020**, *49*. https://doi.org/10.5216/rpt.v49i3.62681.

46. Ortiz Pineda, C.; Temesgen, T.T.; Robertson, L.J. Multiplex quantitative PCR analysis of strawberries from Bogotá, Colombia, for contamination with three parasites. *J. Food Prot.* **2020**, *83*, 1679–1684.

Table S3. Declared federal production of oysters in Santa Catarina, Brazil in 2020. These numbers represent a conservative estimate of the true production as producers are taxed on harvest amounts in federal waters. Values are shown in tons produced and amount of edible product in kilograms (tons * 0.2) calculated using the USDA Handbook on Weights, Measures, and Conversion Factors for Agricultural Commodities and Their Products [77]. Grams per person per day was calculated by dividing the edible weight by the estimated number of adult residents in the Southern region of Brazil that consume shellfish. This figure was estimated by multiplying the proportion of people over 15 (0.789 [78]) by 57.38% (percentage of people who report eating seafood at least once a month [79]) and by the number of residents; 30,192,315 [7].

Seafood type	Tons produced	Edible weight (kg)	g/person/year	g/person/day
Pacific oyster (*Crassostrea gigas*)	2215.5	886200	64.8	0.18
Native oyster (*Crassostrea* spp.)	65.01	26004	1.9	0.005
Total	2280.51	912204	66.7	0.185

References

7. IBGE (Brazilian Institute of Geography and Statistics). Estimates of Resident Population for Municipalities and Federation Units. Available online: https://www.ibge.gov.br/en/statistics/social/population/18448-estimates-of-resident-population-for-municipalities-and-federation-units.html?=&t=resultados (accessed on 7 February 2023).

77. USDA ER. *Weights, Measures, and Conversion Factors for Agricultural Commodities and Their Products*; USDA: Washington, DC, USA, 1992. Available online: http://www.ers.usda.gov/publications/pub-details/?pubid=41881 (accessed on 7 February 2023).

78. Central Intelligence Agency. Brazil. In *The World Factbook*; Central Intelligence Agency: Washington, DC, USA, 2023. Available online: https://www.cia.gov/the-world-factbook/countries/brazil (accessed on 7 February 2023).

79. Baptista, R.C.; Rodrigues, H.; Sant'Ana, A.S. Consumption, knowledge, and food safety practices of Brazilian seafood consumers. *Food Res. Int.* **2020**, *132*, 109084.

Figure S1. Sensitivity analysis visualized through tornado plots for model parameters in Bayesian QRA model. Red indicates the number of *T. gondii* infections/1000 per day using the lower range of a parameter while blue indicates the number of *T. gondii* infections/1000 per day using the upper range of the parameter.

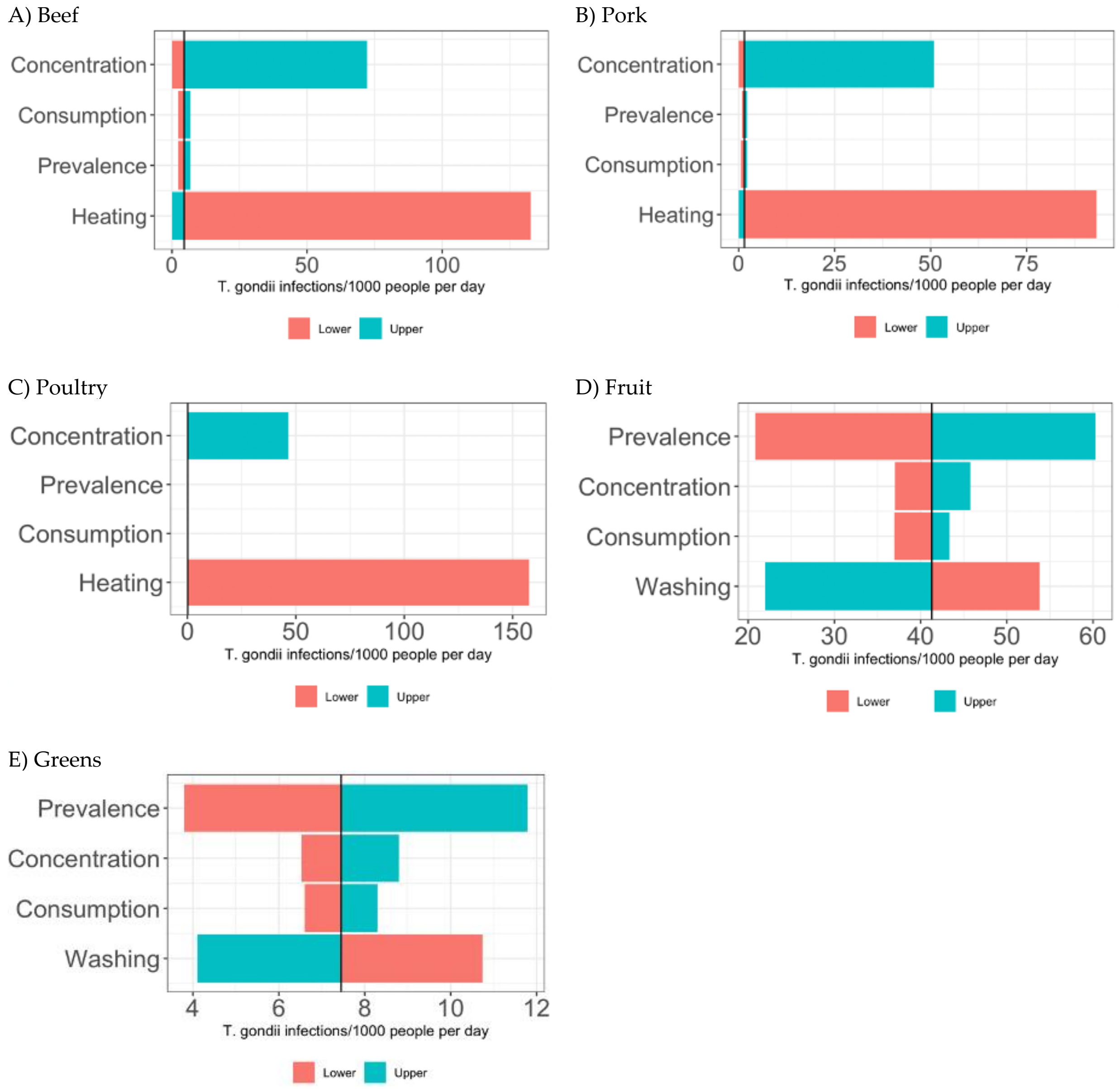

Figure S2. Scenario analysis results for putative scenarios of interest. Food consumption was averaged over all five regions of Brazil to home in on simulated change due to parameter assumptions. Baseline: consumption of food products averaged across all regions of Brazil. Bivalve: Shellfish consumption added as an additional source of foodborne exposure to *T. gondii.* Low beef: Bradyzoite concentration lowered to 1/100 compared to that of other meat products. Low oocyst viability: Oocyst viability halved from baseline to 25%. More washing: Washing produce removed an additional 25% of oocysts compared with baseline.

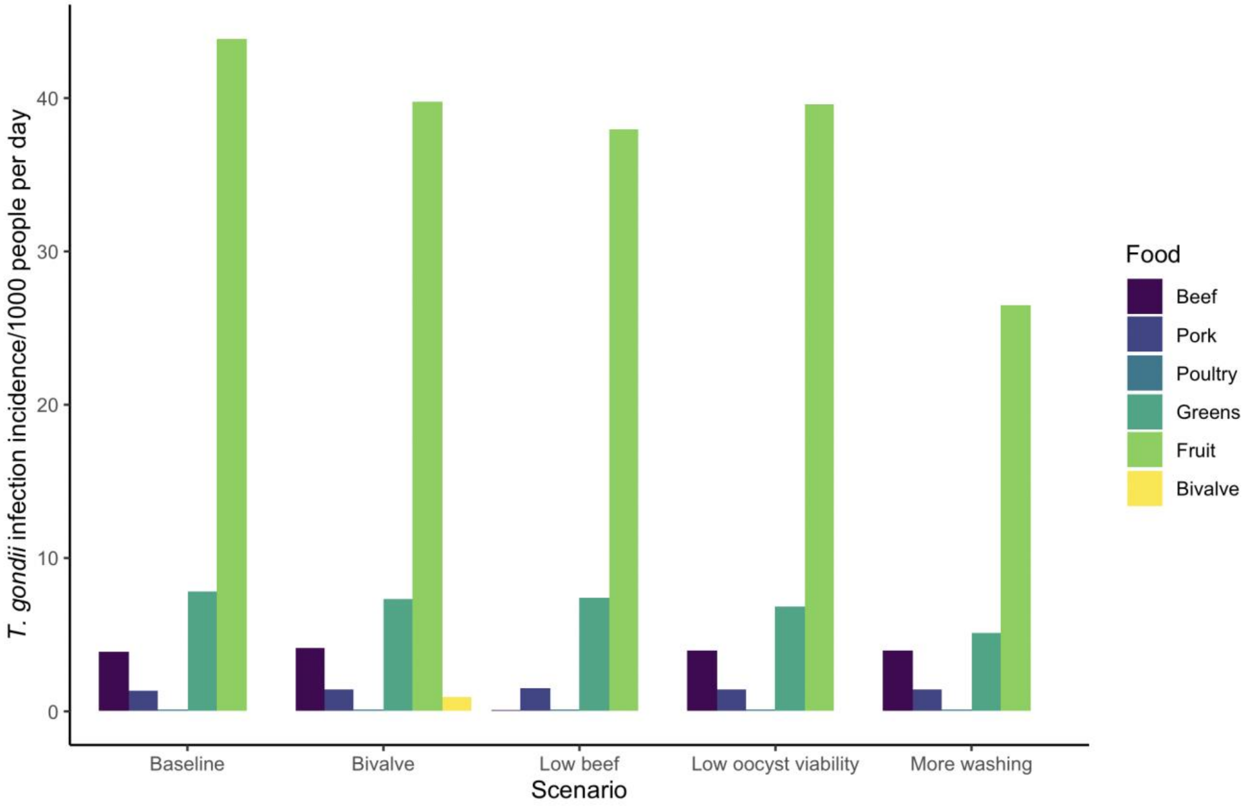

Figure S3. Sample Gelman plots for Bayesian QRA model for the Northern region of Brazil, indicating lack of autocorrelation and presence of convergence.

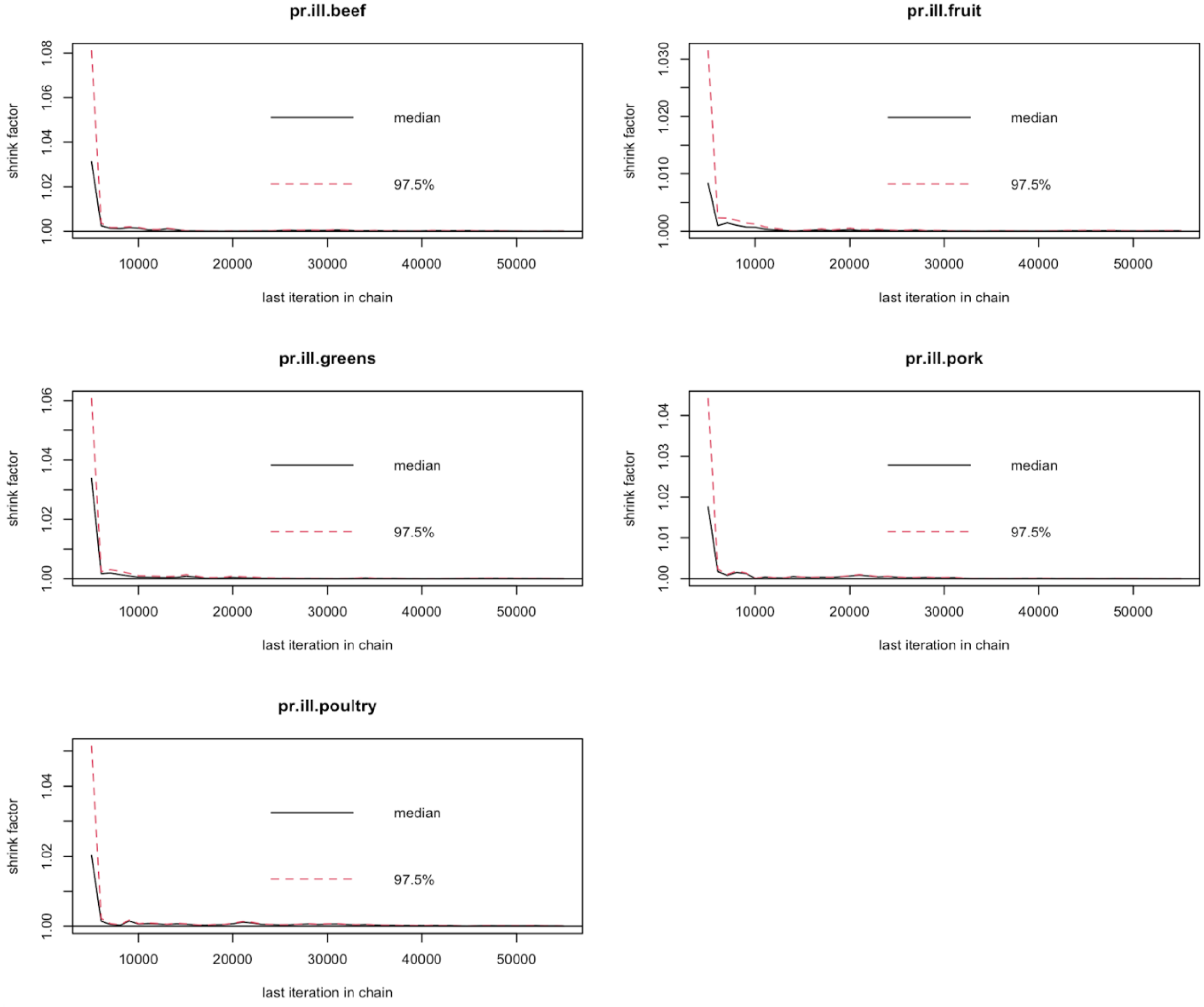

Figure S4. Sample trace plots for Bayesian QRA model for the Northern region of Brazil, indicating adequate mixing and presence of convergence.

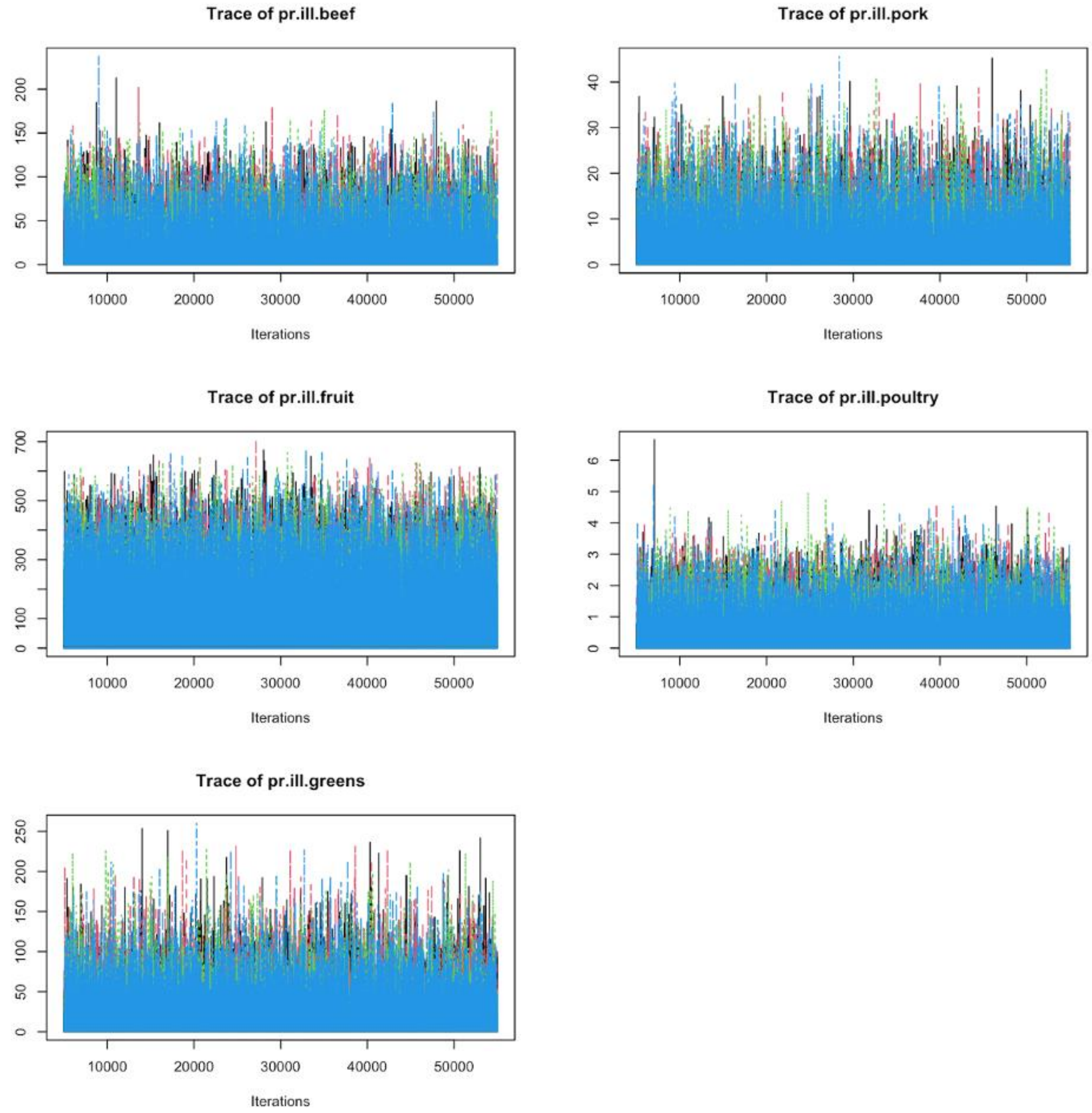

Supplemental Code

```
---
title: "brasilrisksourcecode"
author: "Sophie Zhu"
date: "2021-09-21"
```

````
output: html_document
---
```{r setup, include=FALSE}
knitr::opts_chunk$set(echo = TRUE)
library(ggplot2)
library(mc2d)
library(tidyverse)
library(rjags)
library(ExtDist)
library(gridExtra)
library(MCMCvis)
library(coda)
library(viridis)
library(ggtext)
```

```{r fit oocyst concentration and transform bradyzoite concentration, include=FALSE}
library(fitdistrplus)
library(logspline)

#https://parasitesandvectors.biomedcentral.com/articles/10.1186/s13071-020-04040-2/tables/1
oocystconcentration<-c(1.3, 3.6, 2.8, 8.6, 0.6, 1.1, 3.9, 44.5, 3.2, 6, 38.3, 179.9, 2.5, 3.1, 53.3)

#very skewed beta
descdist(oocystconcentration, discrete=FALSE)

#convert oocyst concentration to proportion out of 180
oocystconcentration180<-oocystconcentration/180

descdist(oocystconcentration180, discrete=FALSE) #, boot=1000)
fit.beta<-fitdist(oocystconcentration180, "beta", method="mle")
fit.betamme<-fitdist(oocystconcentration180, "beta", method="mme")
plot(fit.beta)
summary(fit.beta) #AIC is -31.56
summary(fit.betamme) #AIC is -22.34

summary(rbeta(10000, 0.105, 0.702)*180) #MME median and mean are more conservative
summary(rbeta(10000, 1, 0.446)*180)

#Oocyst washing, proportion between 0 and 100%
oocystwash<-1/100*c(68.61, 52.97,       27.41, 11.41, 34.88, 97.22,  94.17, 97.91, 96.63, 96.12)
descdist(oocystwash, discrete=FALSE)
wash.beta<-fitdist(oocystwash, "beta", method="mme")
plot(wash.beta)
summary(wash.beta)

fit.unif<-fitdist(oocystwash, "unif", method="mme")
plot(fit.unif)
#beta distribution has lower AIC -1.90 compared to uniform -0.95
summary(fit.unif)
summary(rbeta(10000, 0.77, 0.367))
summary(oocystwash)

#bradyzoite concentration used by Opsteegh et al. 2011 and Deng et al. 2020 is a generalized beta with dBeta_ab(6.5,
    5.7, 0, 6.8). However this distribution is not available within rjags. Use normalization to convert generalized beta
    to beta where Z=N-a/b-a
````
````

````
A<-(rBeta_ab(1000, 6.5, 5.7, 0, 6.8))
#https://rdrr.io/cran/prevalence/man/betaPERT.html
B<-(rBeta(1000, 0.955, 0.838)*6.8)

#median 3.6, mean 3.6, max 6.4
summary(rBeta_ab(1000, 6.5, 5.7, 0, 6.8))
#median 3.6, mean 3.59, max 6.7
summary(rBeta(1000, 0.955, 0.838)*6.8)
```

```{r reduction factor, include=FALSE}
#code adapted from Deng et al. 2019 https://www.sciencedirect.com/science/article/pii/S2352352219300040#tbl0001
library(ExtDist)
# reduction factor
calc.heat.reduction <- function( m, s, n ){
  r_geo <- 0.011 # mice
  d.start.heating <- d_inf.heating(p_inf.heating(temp = 25), r = r_geo)
  cooking.temp<- rLaplace(n=n,m=m,s=s)
  cooking.temp<- ifelse( cooking.temp < 25, 25, cooking.temp )
  prob.heat<- p_inf.heating( temp = cooking.temp )
  d_heat<- d_inf.heating( p_heat = prob.heat, r = r_geo )
  rf_heat<- d_heat / d.start.heating

  if( any( is.infinite( rf_heat ) ))
  stop()
  return( rf_heat )
}

#risk of infection in mice
p_inf.heating <- function ( temp ){
1 / (1 + exp( - ( 44.181 - 0.834 * temp )))
}

#number of bradyzoites with above risk
d_inf.heating <- function ( p_heat, r ){ - (log( 1 - p_heat ) / r)
}
```

#####Baseline#####
```{r Bayesian North code, include = TRUE, echo = FALSE}
cat(
"model {
#bradyzoite concentration
bradyzoite.conc~dbeta(0.955, 0.838)

#produce oocyst concentration
oocyst.conc~dbeta(0.105, 0.702)
#c1~dbeta(0.097, 0.66)

#mean amount consumed
beef.serv=58.6
pork.serv=15.3
poultry.serv=51.8
greens.serv=12.3
fruit.serv=45.4
````

```
#bradyzoite or oocyst/consumed amount/food type
lambda.beef <-((10^(bradyzoite.conc*6.8))/100)*beef.serv
beef.brady.raw~dpois(lambda.beef)

lambda.pork <-((10^(bradyzoite.conc*6.8))/100)*pork.serv
pork.brady.raw~dpois(lambda.pork)

lambda.poultry <-((10^(bradyzoite.conc*6.8))/100)*poultry.serv
poultry.brady.raw~dpois(lambda.poultry)

lambda.fruit <-oocyst.conc*180*fruit.serv
fruit.oocyst.raw~dpois(lambda.fruit)

lambda.greens <-oocyst.conc*180*greens.serv
greens.oocyst.raw~dpois(lambda.greens)

#lambda.water <-water.oocystconc*water.serv
#water.oocyst.raw~dpois(lambda.water)

#lambda.shell <-oocyst.conc*shellfish.serv
#shell.oocyst.raw~dpois(lambda.shell)

#######HEATING
#heating temperature distribution
#tempbeef~dLaplace(61.11, 9.82)
tempbeef~ddexp(61.11, 9.82)
temppork~ddexp(61.11, 9.88)
temppoultry~ddexp(65.56, 9.31)

tempbeef.1 <- ifelse(tempbeef < 25, 25, tempbeef)
temppork.1 <- ifelse(temppork < 25, 25, temppork)
temppoultry.1 <- ifelse(temppoultry < 25, 25, temppoultry)

Pbeef<- 1 / (1 + exp( - (44.181 - 0.834 * tempbeef.1)))
Ppork<- 1 / (1 + exp( - (44.181 - 0.834 * temppork.1)))
Ppoultry<- 1 / (1 + exp( - (44.181 - 0.834 * temppoultry.1)))
P25<-1 / (1 + exp( - (44.181 - 0.834 * 25)))

#bradyzoite dose

dose.beef<- -(log(1-Pbeef)/0.011)
dose.pork<- -(log(1-Ppork)/0.011)
dose.poultry<- -(log(1-Ppoultry)/0.011)

dose.25<- -(log(1-P25)/0.011)

#######WASHING
#oocyst removal from washing for 1 minute under running water
washing~dbeta(1.11, 0.53)

#######OOCYST VIABILITY
viability~dunif(0.05, 0.20)

#######Bradyzoite and oocyst concentration post processing

beef.brady<-beef.brady.raw * (dose.beef/dose.25)
```

```
pork.brady<-pork.brady.raw * (dose.pork/dose.25)
poultry.brady<-poultry.brady.raw * (dose.poultry/dose.25)
fruit.oocyst<-fruit.oocyst.raw * (1-washing) * viability
greens.oocyst<-greens.oocyst.raw * (1-washing) * viability

#Dose-response
#single hit probability bradyzoite
r <-0.001535
#single hit probability oocyst
r2 <-0.46

pr.infection.beef <- 1-(exp(-r*beef.brady))
pr.infection.pork <- 1-exp(-r*pork.brady)
pr.infection.poultry <- 1-exp(-r*poultry.brady)
pr.infection.fruit <- 1-exp(-r2*fruit.oocyst)
pr.infection.greens <- 1-exp(-r2*greens.oocyst)
#pr.infection.water <- 1-exp(-r2*water.oocyst.raw)
#pr.infection.shell <- 1-exp(-r2*shell.oocyst.raw)

#######RISK CHARACTERIZATION

#Food prevalence or seroprevalence
beef.prevalence~dbeta(0.74, 1.90)
pork.prevalence~dbeta(1.12, 3.87)
poultry.prevalence~dbeta(1.19, 2.14)
fruit.prevalence~dbeta(0.66, 3.56)
greens.prevalence~dbeta(0.7, 19.9)

#Human seroprevalence
human.prev=0.286

#outcomes of interest
pr.ill.beef <-beef.prevalence*pr.infection.beef*(1-human.prev)*1000
pr.ill.pork <-pork.prevalence*pr.infection.pork*(1-human.prev)*1000
pr.ill.poultry <-poultry.prevalence*pr.infection.poultry*(1-human.prev)*1000
pr.ill.fruit <-fruit.prevalence*pr.infection.fruit*(1-human.prev)*1000
pr.ill.greens <-greens.prevalence*pr.infection.greens*(1-human.prev)*1000
#pr.ill.water <-prev6*pr.infection.water*(1-human.prev)*1000
#pr.ill.shell <-shellfish.prevalence*pr.infection.shell*(1-human.prev)*1000

}", file="brazil.txt")

#specify parameters to be monitored
n.burn= 5000
jagsModel = jags.model(file = "brazil.txt", n.chains = 4, n.adapt = 10000, inits = list(.RNG.name = "base::Wichmann-
    Hill", .RNG.seed = 1989))
params<-c("pr.ill.beef", "pr.ill.pork", "pr.ill.poultry", "pr.ill.fruit", "pr.ill.greens")
update(jagsModel, n.burn)
samps_north<-coda.samples(jagsModel, params, n.iter=50000)

plot(samps_north) #trace of chain and density
gelman.plot(samps_north) #BGR or Gelman-Rubin statistic
#autocorr.plot(samps) #autocorrelation

#gelman.diag(samps)
#autocorr.diag(samps)
```

````
#effectiveSize(samps)
```
```{r Bayesian Northeast code, include = TRUE, echo = FALSE}

cat(
"model {
#bradyzoite concentration
bradyzoite.conc~dbeta(0.955, 0.838)

#produce oocyst concentration
oocyst.conc~dbeta(0.105, 0.702)
#c1~dbeta(0.097, 0.66)

#serving size mean, SD
beef.serv=41.5#~dnorm(41.5, 15)
pork.serv=15.1#~dnorm(15.1, 7.8)
poultry.serv=58.7#~dnorm(58.7, 9.6)
greens.serv=14#~dnorm(14.1, 10.3)
fruit.serv=1.6#~dnorm(1.8, 19.7)

#bradyzoite or oocyst/consumed amount/food type
lambda.beef <-((10^(bradyzoite.conc*6.8))/100)*beef.serv
beef.brady.raw~dpois(lambda.beef)

lambda.pork <-((10^(bradyzoite.conc*6.8))/100)*pork.serv
pork.brady.raw~dpois(lambda.pork)

lambda.poultry <-((10^(bradyzoite.conc*6.8))/100)*poultry.serv
poultry.brady.raw~dpois(lambda.poultry)

lambda.fruit <-oocyst.conc*180*fruit.serv
fruit.oocyst.raw~dpois(lambda.fruit)

lambda.greens <-oocyst.conc*180*greens.serv
greens.oocyst.raw~dpois(lambda.greens)

#######HEATING
#heating temperature distribution
#tempbeef~dLaplace(61.11, 9.82)
tempbeef~ddexp(61.11, 9.82)
temppork~ddexp(61.11, 9.88)
temppoultry~ddexp(65.56, 9.31)

tempbeef.1 <- ifelse(tempbeef < 25, 25, tempbeef)
temppork.1 <- ifelse(temppork < 25, 25, temppork)
temppoultry.1 <- ifelse(temppoultry < 25, 25, temppoultry)

Pbeef<- 1 / (1 + exp( - (44.181 - 0.834 * tempbeef.1)))
Ppork<- 1 / (1 + exp( - (44.181 - 0.834 * temppork.1)))
Ppoultry<- 1 / (1 + exp( - (44.181 - 0.834 * temppoultry.1)))
P25<-1 / (1 + exp( - (44.181 - 0.834 * 25)))

#bradyzoite dose

dose.beef<- -(log(1-Pbeef)/0.011)
dose.pork<- -(log(1-Ppork)/0.011)
````

```
dose.poultry<- -(log(1-Ppoultry)/0.011)

dose.25<- -(log(1-P25)/0.011)

#######WASHING
#oocyst removal from washing for 1 minute under running water
washing~dbeta(1.11, 0.53)

#######OOCYST VIABILITY
viability~dunif(0.05, 0.20)

#######Bradyzoite and oocyst concentration post processing

beef.brady<-beef.brady.raw * (dose.beef/dose.25)
pork.brady<-pork.brady.raw * (dose.pork/dose.25)
poultry.brady<-poultry.brady.raw * (dose.poultry/dose.25)
fruit.oocyst<-fruit.oocyst.raw * (1-washing) * viability
greens.oocyst<-greens.oocyst.raw * (1-washing) * viability

#Dose-response
#single hit probability bradyzoite
r <-0.001535
#single hit probability oocyst
r2 <-0.46

pr.infection.beef <- 1-(exp(-r*beef.brady))
pr.infection.pork <- 1-exp(-r*pork.brady)
pr.infection.poultry <- 1-exp(-r*poultry.brady)
pr.infection.fruit <- 1-exp(-r2*fruit.oocyst)
pr.infection.greens <- 1-exp(-r2*greens.oocyst)

#######RISK CHARACTERIZATION

#Food prevalence or seroprevalence
beef.prevalence~dbeta(0.74, 1.90)
pork.prevalence~dbeta(1.12, 3.87)
poultry.prevalence~dbeta(1.19, 2.14)
fruit.prevalence~dbeta(0.66, 3.56)
greens.prevalence~dbeta(0.7, 19.9)

#Human seroprevalence
human.prev=0.286

pr.ill.beef <-beef.prevalence*pr.infection.beef*(1-human.prev)*1000
pr.ill.pork <-pork.prevalence*pr.infection.pork*(1-human.prev)*1000
pr.ill.poultry <-poultry.prevalence*pr.infection.poultry*(1-human.prev)*1000
pr.ill.fruit <-fruit.prevalence*pr.infection.fruit*(1-human.prev)*1000
pr.ill.greens <-greens.prevalence*pr.infection.greens*(1-human.prev)*1000

}", file="brazil.txt")

#specify parameters to be monitored
n.burn= 5000
jagsModel = jags.model(file = "brazil.txt", n.chains = 4, n.adapt = 10000, inits = list(.RNG.name = "base::Wichmann-
    Hill", .RNG.seed = 1989))
params<-c("pr.ill.beef", "pr.ill.pork", "pr.ill.poultry", "pr.ill.fruit", "pr.ill.greens")
```

```
update(jagsModel, n.burn)
samps_northeast<-coda.samples(jagsModel, params, n.iter=50000)
```
```{r Bayesian Southeast code, include = TRUE, echo = FALSE}

cat(
"model {
#bradyzoite concentration
bradyzoite.conc~dbeta(0.955, 0.838)

#produce oocyst concentration
oocyst.conc~dbeta(0.105, 0.702)
#c1~dbeta(0.097, 0.66)

#serving size mean, SD
beef.serv=48.4#~dnorm(50.8, 15)
pork.serv=20.3#~dnorm(29.2, 7.8)
poultry.serv=44.9#~dnorm(37.4, 9.6)
fruit.serv=1.1#~dnorm(1.7, 19.7)
greens.serv=28.8#~dnorm(24.4, 10.3)

#bradyzoite or oocyst/consumed amount/food type
lambda.beef <-((10^(bradyzoite.conc*6.8))/100)*beef.serv
beef.brady.raw~dpois(lambda.beef)

lambda.pork <-((10^(bradyzoite.conc*6.8))/100)*pork.serv
pork.brady.raw~dpois(lambda.pork)

lambda.poultry <-((10^(bradyzoite.conc*6.8))/100)*poultry.serv
poultry.brady.raw~dpois(lambda.poultry)

lambda.fruit <-oocyst.conc*180*fruit.serv
fruit.oocyst.raw~dpois(lambda.fruit)

lambda.greens <-oocyst.conc*180*greens.serv
greens.oocyst.raw~dpois(lambda.greens)

#######HEATING
#heating temperature distribution
#tempbeef~dLaplace(61.11, 9.82)
tempbeef~ddexp(61.11, 9.82)
temppork~ddexp(61.11, 9.88)
temppoultry~ddexp(65.56, 9.31)

tempbeef.1 <- ifelse(tempbeef < 25, 25, tempbeef)
temppork.1 <- ifelse(temppork < 25, 25, temppork)
temppoultry.1 <- ifelse(temppoultry < 25, 25, temppoultry)

Pbeef<- 1 / (1 + exp( - (44.181 - 0.834 * tempbeef.1)))
Ppork<- 1 / (1 + exp( - (44.181 - 0.834 * temppork.1)))
Ppoultry<- 1 / (1 + exp( - (44.181 - 0.834 * temppoultry.1)))
P25<-1 / (1 + exp( - (44.181 - 0.834 * 25)))

#bradyzoite dose

dose.beef<- -(log(1-Pbeef)/0.011)
```

```
dose.pork<- -(log(1-Ppork)/0.011)
dose.poultry<- -(log(1-Ppoultry)/0.011)

dose.25<- -(log(1-P25)/0.011)

#######WASHING
#oocyst removal from washing for 1 minute under running water
washing~dbeta(1.11, 0.53)

#######OOCYST VIABILITY
viability~dunif(0.05, 0.20)

#######Bradyzoite and oocyst concentration post processing

beef.brady<-beef.brady.raw * (dose.beef/dose.25)
pork.brady<-pork.brady.raw * (dose.pork/dose.25)
poultry.brady<-poultry.brady.raw * (dose.poultry/dose.25)
fruit.oocyst<-fruit.oocyst.raw * (1-washing) * viability
greens.oocyst<-greens.oocyst.raw * (1-washing) * viability

#Dose-response
#single hit probability bradyzoite
r <-0.001535
#single hit probability oocyst
r2 <-0.46

pr.infection.beef <- 1-(exp(-r*beef.brady))
pr.infection.pork <- 1-exp(-r*pork.brady)
pr.infection.poultry <- 1-exp(-r*poultry.brady)
pr.infection.fruit <- 1-exp(-r2*fruit.oocyst)
pr.infection.greens <- 1-exp(-r2*greens.oocyst)

#######RISK CHARACTERIZATION

#Food prevalence or seroprevalence
beef.prevalence~dbeta(0.74, 1.90)
pork.prevalence~dbeta(1.12, 3.87)
poultry.prevalence~dbeta(1.19, 2.14)
fruit.prevalence~dbeta(0.66, 3.56)
greens.prevalence~dbeta(0.7, 19.9)

#Human seroprevalence
human.prev=0.286

pr.ill.beef <-beef.prevalence*pr.infection.beef*(1-human.prev)*1000
pr.ill.pork <-pork.prevalence*pr.infection.pork*(1-human.prev)*1000
pr.ill.poultry <-poultry.prevalence*pr.infection.poultry*(1-human.prev)*1000
pr.ill.fruit <-fruit.prevalence*pr.infection.fruit*(1-human.prev)*1000
pr.ill.greens <-greens.prevalence*pr.infection.greens*(1-human.prev)*1000

}", file="brazil.txt")

#specify parameters to be monitored
n.burn= 5000
jagsModel = jags.model(file = "brazil.txt", n.chains = 4, n.adapt = 10000, inits = list(.RNG.name = "base::Wichmann-
    Hill", .RNG.seed = 1989))
```

````
params<-c("pr.ill.beef", "pr.ill.pork", "pr.ill.poultry", "pr.ill.fruit", "pr.ill.greens")
update(jagsModel, n.burn)
samps_southeast<-coda.samples(jagsModel, params, n.iter=50000)
```

```{r Bayesian South code, include = TRUE, echo = FALSE}
cat(
"model {
#bradyzoite concentration
bradyzoite.conc~dbeta(0.955, 0.838)

#produce oocyst concentration
oocyst.conc~dbeta(0.105, 0.702)
#c1~dbeta(0.097, 0.66)

#serving size mean, SD
beef.serv=50.8#~dnorm(80.7, 15)
pork.serv=29.5#~dnorm(31.6, 7.8)
poultry.serv=37.4#~dnorm(35.8, 9.6)
fruit.serv=0.4#~dnorm(3.1, 19.7)
greens.serv=22.5#~dnorm(37.7, 10.3)

#bradyzoite or oocyst/consumed amount/food type
lambda.beef <-((10^(bradyzoite.conc*6.8))/100)*beef.serv
beef.brady.raw~dpois(lambda.beef)

lambda.pork <-((10^(bradyzoite.conc*6.8))/100)*pork.serv
pork.brady.raw~dpois(lambda.pork)

lambda.poultry <-((10^(bradyzoite.conc*6.8))/100)*poultry.serv
poultry.brady.raw~dpois(lambda.poultry)

lambda.fruit <-oocyst.conc*180*fruit.serv
fruit.oocyst.raw~dpois(lambda.fruit)

lambda.greens <-oocyst.conc*180*greens.serv
greens.oocyst.raw~dpois(lambda.greens)


#######HEATING
#heating temperature distribution
#tempbeef~dLaplace(61.11, 9.82)
tempbeef~ddexp(61.11, 9.82)
temppork~ddexp(61.11, 9.88)
temppoultry~ddexp(65.56, 9.31)

tempbeef.1 <- ifelse(tempbeef < 25, 25, tempbeef)
temppork.1 <- ifelse(temppork < 25, 25, temppork)
temppoultry.1 <- ifelse(temppoultry < 25, 25, temppoultry)


Pbeef<- 1 / (1 + exp( - (44.181 - 0.834 * tempbeef.1)))
Ppork<- 1 / (1 + exp( - (44.181 - 0.834 * temppork.1)))
Ppoultry<- 1 / (1 + exp( - (44.181 - 0.834 * temppoultry.1)))
P25<-1 / (1 + exp( - (44.181 - 0.834 * 25)))
````

```
#bradyzoite dose

dose.beef<- -(log(1-Pbeef)/0.011)
dose.pork<- -(log(1-Ppork)/0.011)
dose.poultry<- -(log(1-Ppoultry)/0.011)

dose.25<- -(log(1-P25)/0.011)

#if( any( is.infinite(dose.beef) ))
#stop()
#return(dose.beef)

#######WASHING
#oocyst removal from washing for 1 minute under running water
washing~dbeta(1.11, 0.53)

#######OOCYST VIABILITY
viability~dunif(0.05, 0.20)

#######Bradyzoite and oocyst concentration post processing

beef.brady<-beef.brady.raw * (dose.beef/dose.25)
pork.brady<-pork.brady.raw * (dose.pork/dose.25)
poultry.brady<-poultry.brady.raw * (dose.poultry/dose.25)
fruit.oocyst<-fruit.oocyst.raw * (1-washing) * viability
greens.oocyst<-greens.oocyst.raw * (1-washing) * viability

#Dose-response
#single hit probability bradyzoite
r <-0.001535
#single hit probability oocyst
r2 <-0.46

pr.infection.beef <- 1-(exp(-r*beef.brady))
pr.infection.pork <- 1-exp(-r*pork.brady)
pr.infection.poultry <- 1-exp(-r*poultry.brady)
pr.infection.fruit <- 1-exp(-r2*fruit.oocyst)
pr.infection.greens <- 1-exp(-r2*greens.oocyst)

#######RISK CHARACTERIZATION

#Food prevalence or seroprevalence
beef.prevalence~dbeta(0.74, 1.90)
pork.prevalence~dbeta(1.12, 3.87)
poultry.prevalence~dbeta(1.19, 2.14)
fruit.prevalence~dbeta(0.66, 3.56)
greens.prevalence~dbeta(0.7, 19.9)

#Human seroprevalence
human.prev=0.286

pr.ill.beef <-beef.prevalence*pr.infection.beef*(1-human.prev)*1000
pr.ill.pork <-pork.prevalence*pr.infection.pork*(1-human.prev)*1000
pr.ill.poultry <-poultry.prevalence*pr.infection.poultry*(1-human.prev)*1000
pr.ill.fruit <-fruit.prevalence*pr.infection.fruit*(1-human.prev)*1000
pr.ill.greens <-greens.prevalence*pr.infection.greens*(1-human.prev)*1000
```

```
}", file="brazil.txt")

#specify parameters to be monitored
n.burn= 5000
jagsModel = jags.model(file = "brazil.txt", n.chains = 4, n.adapt = 10000, inits = list(.RNG.name = "base::Wichmann-
        Hill", .RNG.seed = 1989))
params<-c("pr.ill.beef", "pr.ill.pork", "pr.ill.poultry", "pr.ill.fruit", "pr.ill.greens")
update(jagsModel, n.burn)
samps_south<-coda.samples(jagsModel, params, n.iter=50000)
```

```{r Bayesian Centerwest code, include = TRUE, echo = FALSE}
cat(
"model {
#bradyzoite concentration
bradyzoite.conc~dbeta(0.955, 0.838)

#produce oocyst concentration
oocyst.conc~dbeta(0.105, 0.702)
#c1~dbeta(0.097, 0.66)

#serving size mean, SD
beef.serv=80.7
pork.serv=31.5
poultry.serv=35.8
fruit.serv=2.6
greens.serv=37.7

#bradyzoite or oocyst/consumed amount/food type
lambda.beef <-((10^(bradyzoite.conc*6.8))/100)*beef.serv
beef.brady.raw~dpois(lambda.beef)

lambda.pork <-((10^(bradyzoite.conc*6.8))/100)*pork.serv
pork.brady.raw~dpois(lambda.pork)

lambda.poultry <-((10^(bradyzoite.conc*6.8))/100)*poultry.serv
poultry.brady.raw~dpois(lambda.poultry)

lambda.fruit <-oocyst.conc*180*fruit.serv
fruit.oocyst.raw~dpois(lambda.fruit)

lambda.greens <-oocyst.conc*180*greens.serv
greens.oocyst.raw~dpois(lambda.greens)

#######HEATING
#heating temperature distribution
#tempbeef~dLaplace(61.11, 9.82)
tempbeef~ddexp(61.11, 9.82)
temppork~ddexp(61.11, 9.88)
temppoultry~ddexp(65.56, 9.31)

tempbeef.1 <- ifelse(tempbeef < 25, 25, tempbeef)
temppork.1 <- ifelse(temppork < 25, 25, temppork)
temppoultry.1 <- ifelse(temppoultry < 25, 25, temppoultry)
```

```
Pbeef<- 1 / (1 + exp( - (44.181 - 0.834 * tempbeef.1)))
Ppork<- 1 / (1 + exp( - (44.181 - 0.834 * temppork.1)))
Ppoultry<- 1 / (1 + exp( - (44.181 - 0.834 * temppoultry.1)))
P25<-1 / (1 + exp( - (44.181 - 0.834 * 25)))

#bradyzoite dose

dose.beef<- -(log(1-Pbeef)/0.011)
dose.pork<- -(log(1-Ppork)/0.011)
dose.poultry<- -(log(1-Ppoultry)/0.011)

dose.25<- -(log(1-P25)/0.011)

#######WASHING
#oocyst removal from washing for 1 minute under running water
washing~dbeta(1.11, 0.53)

#######OOCYST VIABILITY
viability~dunif(0.05, 0.20)

#######Bradyzoite and oocyst concentration post processing

beef.brady<-beef.brady.raw * (dose.beef/dose.25)
pork.brady<-pork.brady.raw * (dose.pork/dose.25)
poultry.brady<-poultry.brady.raw * (dose.poultry/dose.25)
fruit.oocyst<-fruit.oocyst.raw * (1-washing) * viability
greens.oocyst<-greens.oocyst.raw * (1-washing) * viability

#Dose-response
#single hit probability bradyzoite
r <-0.001535
#single hit probability oocyst
r2 <-0.46

pr.infection.beef <- 1-(exp(-r*beef.brady))
pr.infection.pork <- 1-exp(-r*pork.brady)
pr.infection.poultry <- 1-exp(-r*poultry.brady)
pr.infection.fruit <- 1-exp(-r2*fruit.oocyst)
pr.infection.greens <- 1-exp(-r2*greens.oocyst)

#######RISK CHARACTERIZATION

#Food prevalence or seroprevalence
beef.prevalence~dbeta(0.74, 1.90)
pork.prevalence~dbeta(1.12, 3.87)
poultry.prevalence~dbeta(1.19, 2.14)
fruit.prevalence~dbeta(0.66, 3.56)
greens.prevalence~dbeta(0.7, 19.9)

#Human seroprevalence
human.prev=0.286

pr.ill.beef <-beef.prevalence*pr.infection.beef*(1-human.prev)*1000
pr.ill.pork <-pork.prevalence*pr.infection.pork*(1-human.prev)*1000
pr.ill.poultry <-poultry.prevalence*pr.infection.poultry*(1-human.prev)*1000
pr.ill.fruit <-fruit.prevalence*pr.infection.fruit*(1-human.prev)*1000
```

```
pr.ill.greens <-greens.prevalence*pr.infection.greens*(1-human.prev)*1000
}", file="brazil.txt")

#specify parameters to be monitored
n.burn= 5000
jagsModel = jags.model(file = "brazil.txt", n.chains = 4, n.adapt = 10000, inits = list(.RNG.name = "base::Wichmann-
        Hill", .RNG.seed = 1989))
params<-c("pr.ill.beef", "pr.ill.pork", "pr.ill.poultry", "pr.ill.fruit", "pr.ill.greens")
update(jagsModel, n.burn)
samps_centerwest<-coda.samples(jagsModel, params, n.iter=50000)
```

###Baseline plot###

```{r Baseline Plot, include=TRUE, echo=FALSE}
summary(samps_north)
summary(samps_northeast)
summary(samps_southeast)
summary(samps_south)
summary(samps_centerwest)

output<-data.frame(
  region=c("North", "Northeast", "Southeast", "South", "Centerwest"),
  Beef=c(4.81, 3.78, 6.32, 5.19, 3.58),
  Pork=c(0.97, 1.28, 1.91, 1.91, 2.03),
  Poultry=c(0.13, 0.17, 0.11, 0.15, 0.11),
  Greens=c(7.69, 7.58, 8.86, 9.68, 9.67),
  Fruit=c(48.13, 24.62, 20.31, 17.85, 11.76),
  bivalve=c(NA, NA, NA, NA, NA)
)

output<-reshape2::melt(output, id.vars = "region", variable.name="Food", value.name = "incidence")

output$region<-as.factor(output$region)

ggplot(output, aes(x = region, y= incidence, fill = Food)) +
  geom_bar(position="dodge", stat = "identity") + theme_classic() + ylab("*T. gondii* infection incidence/1000 people per
        day") + xlab("Region") + scale_fill_viridis(discrete = TRUE, breaks = c('Beef', 'Pork', 'Poultry', 'Greens', 'Fruit'))+
        theme(axis.title.y=ggtext::element_markdown())

A<-MCMCplot(samps_north, ci=c(50, 90))
B<-MCMCplot(samps_northeast)
C<-MCMCplot(samps_southeast)
D<-MCMCplot(samps_south)
E<-MCMCplot(samps_centerwest)

MCMCtrace(samps_north)
MCMCtrace(samps_northeast)
```
